# Association between MRI indicators of the glymphatic system and cognition in high-risk populations for Alzheimer's disease

**DOI:** 10.1016/j.tjpad.2026.100504

**Published:** 2026-02-20

**Authors:** Li Jiang, Ling Zhang, Shu-Xian Wu, Qin-Qin Zhu, Wei Wang, Jia-Wei Gao, Yi Zhu, Shui Tian, Ming Qi

**Affiliations:** a Department of Radiology, The First Affiliated Hospital with Nanjing Medical University, Nanjing 210029, Jiangsu Province, China; b Department of Radiology, Mianyang Central Hospital, School of Medicine, University of Electronic Science and Technology of China, Mianyang, China; c Department of Radiology, Liyang People's Hospital, Changzhou 213300, Jiangsu Province, China

**Keywords:** Subjective cognitive decline, Mild cognitive impairment, Diffusion tensor image analysis, Perivascular space burden, Glymphatic system

## Abstract

**Background:**

Using the Diffusion Tensor Image Analysis Along the Perivascular Space (ALPS) method and perivascular space (PVS) burden to study glymphatic function in high-risk Alzheimer's disease (AD) populations, this research investigates the correlation between ALPS index and PVS volume with cognitive function respectively.

**Methods:**

This study enrolled 126 participants, including 21 cognitively unimpaired (CU) individuals, 68 with subjective cognitive decline (SCD), and 37 with mild cognitive impairment (MCI). All participants underwent MRI and cognitive assessments. MRI measures, including the PVS burden and the ALPS index, were compared across SCD, MCI, and CU groups. Additionally, correlations among the ALPS index, PVS burden, and cognitive scales were analyzed.

**Results:**

The PVS in the basal ganglia volume fraction (PVSVF-BG) in patients with MCI was significantly larger than the fraction in CUs (*p* < 0.05) and a higher PVSVF-BG was associated with poorer performance on the Trail Making Test A (TMTA) (*r* = 0.29, *p* < 0.05). Compared with the CU and SCD groups, patients with MCI exhibited a significantly lower ALPS index in both the left (*p* < 0.05) and right hemispheres (*p* < 0.001). Lower whole brain ALPS index in patients with MCI was correlated with worse performance in the Auditory Verbal Learning Test(AVLT) (N4, *r* = 0.33, *p* < 0.05; N7, *r* = 0.56, *p* < 0.001).

**Conclusions:**

An increased PVS burden and a decreased ALPS index can be observed in the preclinical stage of AD, which may suggest impaired glymphatic system function. These impairments were further correlated with worse cognitive performance in terms of attention in SCD and memory in MCI.

## Highlights

• Glymphatic function characterized by ALPS index becomes abnormal in the early stages of AD.
• The PVS burden in the basal ganglia region becomes abnormal in MCI patients.
• The PVS burden and ALPS index are closely associated with several cognitive scales.

## Graphical abstract

**Figure:**
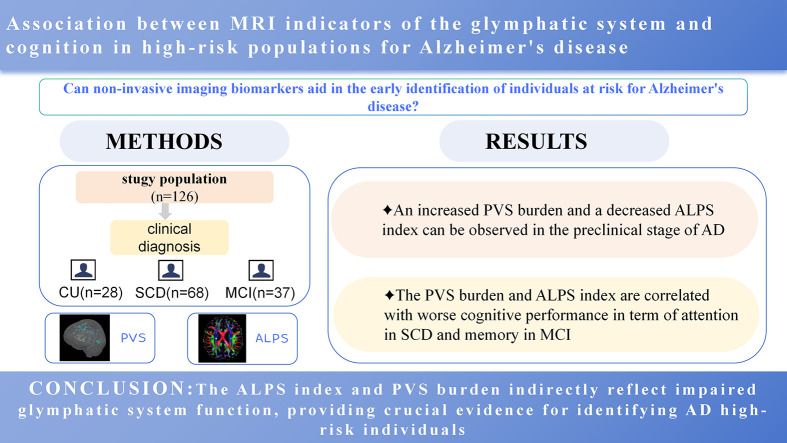

## 1 Introduction

Worldwide, Alzheimer’s disease (AD) and related dementia cases have exceeded 51 million, and is projected to reach 70 million by 2030 [1]. The National Institute on Aging (NIA) and the Alzheimer’s Association define AD as a continuum: subjective cognitive decline (SCD), mild cognitive impairment (MCI), and the dementia stage [2]. Previous studies have demonstrated that pathological changes begin 10-20 years before clinical symptoms [3,4]. Consequently, the SCD and MCI stages present crucial opportunities for therapeutic intervention. This imminent crisis underscores the urgency of identifying reliable biomarkers to detect Alzheimer's disease at its earliest and potentially reversible, or even modifiable, stage. AD is characterized by three core hallmarks, including extracellular β-amyloid (Aβ) plaques, intracellular neurofibrillary tangles, and sustained neuroinflammation [5]. The glymphatic system facilitates the clearance of Aβ and other soluble proteins by enabling cerebrospinal fluid (CSF) to enter the brain parenchyma via arterial perivascular spaces (PVS), clear metabolic waste, and drain through venous pathways [6,7]. Although contrast agent-dependent techniques can accurately reflect glymphatic function, their invasiveness limits clinical applicability. Emerging non-invasive MRI methods, such as diffusion tensor imaging along the perivascular space (ALPS) and PVS metrics, show promise but require further validation of their clinical efficacy [[8], [9], [10]].

According to the Standards for Reporting Vascular Changes on Neuroimaging (STRIVE) criteria, enlarged PVS (EPVS) are fluid-filled structures that follow vascular trajectories through brain tissue, exhibit CSF-like signal intensity on MRI, display round, ovoid, or linear morphology, and typically measure <3 mm in diameter. While present across all ages, they become visible on MRI only when dilated [11]. Previous studies have found that individuals with AD and MCI exhibit significantly higher PVS volumes compared with cognitively unimpaired (CU) participants without SCD [12]. Moreover, PVS burden in the basal ganglia (PVS-BG) is significantly elevated in patients with AD relative to CU individuals [13]. These findings suggest that EPVS burden may contribute to the onset and progression of neurodegenerative disease. Furthermore, the ALPS index, introduced by Taoka et al. [9], quantifies water diffusivity along deep medullary veins adjacent to the lateral ventricles. It has been widely applied in studies of various central nervous system neurodegenerative diseases [[14], [15], [16], [17]]and is recognized as a potential biomarker for AD progression [18,19]. Previous studies consistently report reduced ALPS indices in AD patients, which correlate with the severity of cognitive impairment [[13], [14], [15], [16], [17], [18], [19], [20], [21]].

However, it remains unclear whether EPVS burden and ALPS index can serve as valid biomarkers for SCD and MCI. Therefore, we hypothesize that an increased PVS burden and a decreased ALPS index can be observed in the preclinical stage of AD. We will employ these two indicators to assess glymphatic function across three participant groups and explore its correlation with cognitive performance, aiming to identify early imaging biomarkers for AD.

## 2 Methods and materials

### 2.1 Participants

**Fig. 1:**
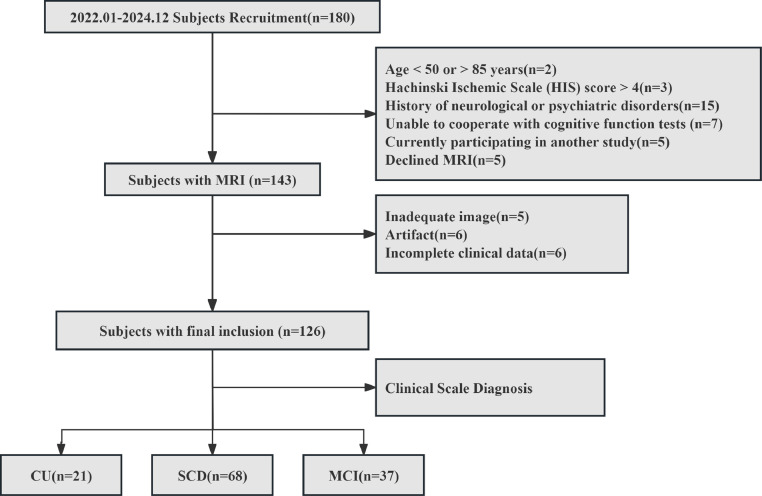
The flowchart of subjects inclusion and exclusion criteria.

This study was reviewed and approved by the local institutional review board (protocol number: 2019-SR-015). All enrolled participants provided written informed consent prior to participation. A total of 126 participants were recruited from the First Affiliated Hospital with Nanjing Medical University between January 2022 and December 2024, comprising 37 patients with MCI, 68 with SCD, and 21 CU individuals (Fig. 1). Enrollment criteria followed established protocols from our research group [22]. Details of the neuropsychological assessments are provided in Supplementary S1(1). Levels of amyloid-β42 (Aβ42), phosphorylated tau (Tau-181), and blood lipids, including total cholesterol (CHOL), triglyceride (TG), high-density lipoprotein (HDL-C), low-density lipoprotein (LDL-C), apolipoprotein B (APOB), and glucose (GLU) were obtained from morning fasting blood samples.

### 2.2 MRI acquisition

Diffusion-weighted (DW), T1-weighted (T1w), and fluid-attenuated inversion recovery (FLAIR) imaging data were acquired for each participant using a 3-T scanner. Additional imaging parameters are detailed in Supplementary S1(2).

### 2.3 Grading of white matter lesions

On FLAIR images, white matter lesions (WMLs) were assessed in the periventricular white matter (PVWM) and deep white matter (DWM) according to the Fazekas rating scale [23]. For each white matter region, participants were rated on a scale from 0 to 3. Additionally, the total Fazekas score was calculated by summing the two regional ratings, yielding a range from 0 to 6.

### 2.4 Qualitative and Quantitative Assessment of EPVS Burden

**Fig. 2:**
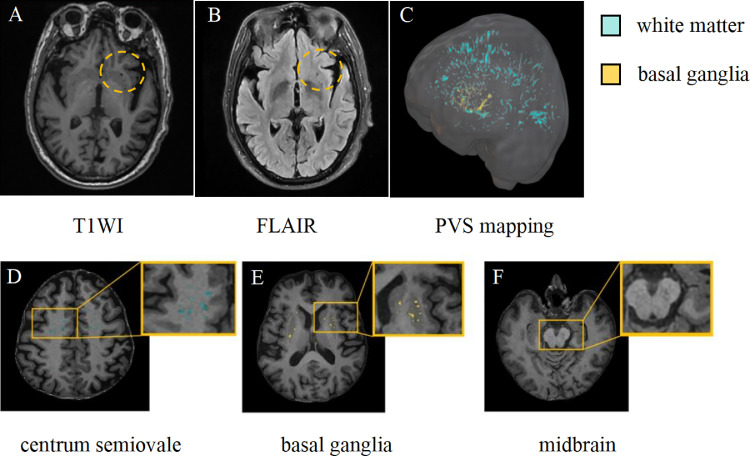
Schematic diagram of PVS Fig. 2 EPVS appears hypointense on both T1WI and FLAIR images (yellow dotted circle) (A–B), PVS maps of the white matter (blue) and basal ganglia (yellow) obtained by applying the Frangi filter for PVS segmentation(C), PVS example images from the centrum semiovale, basal ganglia, and midbrain of a 70-year-old participant(D-F). T1WI, T1-Weighted Imaging; FLAIR, Fluid-Attenuated Inversion Recovery; PVS, perivascular spaces.

Several tools have been developed to analyze PVS as biomarkers of glymphatic system dysfunction. Among the most extensively studied methods are the PVS visual rating scale and automated quantitative measurement of PVS volume. The study comprehensively assessed the presence of PVS using baseline MR imaging with T1w and FLAIR sequences (Fig. 2A-B), while the T1w sequence was specifically employed for EPVS counting. Furthermore, EPVS were quantitatively assessed in the centrum semiovale (CSO), basal ganglia(BG), and midbrain regions [24]. Per the online user guide (http://www.sbirc.ed.ac.uk/documents/epvs-rating-scale-user-guide.pdf), EPVS in the specified region are assessed within the hemisphere and single slice where they are most abundant, and rated on a 5-point scale: 0 (none), 1 (mild, 1–10), 2 (moderate, 11–20), 3 (frequent, 21–40), or 4 (severe, >40). In contrast, midbrain EPVS were evaluated using a binary system: 0 (not visible) or 1 (visible) (Fig. 2D-F). The inter-rater reliability of the visual rating scales was assessed by two experienced radiologists who were blinded to the clinical groups.

The PVS volume was quantified using an automated and highly reliable approach, based on a pipeline established in prior studies [25]. The procedural steps were as follows: T1w and FLAIR images underwent preprocessing with FSL (version 6.0.1, FMRIB Software Library; http://www.fmrib.ox.ac.uk/fsl), which included motion correction, non-uniform intensity normalization, computation of Talairach transformation, global intensity normalization, and skull stripping. Subsequently, non-local means filtering was applied to the T1w and FLAIR images to generate enhanced perivascular space contrast (EPC) images. Segmentation of the MRI images was performed using the n-tissue technique from the ANTs (Advanced Normalization Tools; http://github.com/ANTsX/ANTs) software package [26] to extract masks of WM and BG. These masks were then used to calculate PVS volume. The Frangi filter [27] was applied to T1w, FLAIR, and EPC images using the Quantitative Imaging Toolkit (https://cabeen.io/qitwiki/) to enhance tubular structures. Finally, the PVS volume fraction (PVSVF; defined as PVS volume divided by intracranial volume) was computed to account for inter-individual differences in brain size(Fig. 2C).

### 2.5 ALPS index calculation

The DTI data were preprocessed by using Mrtrix3 software (https://www.mrtrix.org/), including noise correction, gibbs ringing correction, eddy correction, N4 bias field correction, obtain the brain mask image and overlay it with the original image in MRIcron to verify the accuracy of the extraction. Next, using the dtifit function in FSL, calculate the tensor, then extract the diffusion components from the tensor data, generate a color-coded fractional anisotropy (FA) map to visualize fiber orientation, register the data to standard template space, apply reorientation to the tensor data, and finally produce files suitable for ROI analysis in ITK-SNAP software (https://www.itksnap.org/). On a consistent axial slice at the level of the lateral ventricle body, two neuroradiologists blinded to all clinical data manually placed four 6-mm square regions of interest (ROIs) on the color-coded FA map in ITK-SNAP. ROIs were positioned within the blue-coded (projection) and green-coded (association) fiber tracts adjacent to the lateral ventricular wall based on predefined anatomical landmarks. Diffusivity values were then extracted for calculation. The diffusivities along the x-axis (Dxx), y-axis (Dyy), and z-axis (Dzz) of each ROI were recorded, including mean x-axis diffusivity in projection fibers (Dxproj), mean x-axis diffusivity in association fibers (Dxassoc), mean y-axis diffusivity in projection fibers (Dyproj), and mean z-axis diffusivity in association fibers (Dzassoc). Additionally, the DTI-ALPS indices for the left hemisphere, right hemisphere, and whole brain were calculated as shown in the equation. The intraclass correlation coefficient (ICC) was used to assess the inter-observer agreement.

$$\text{ALPS}=\frac{\text{mean}\left(D_{\text{xzproj}},D_{\text{zzassoc}}\right)}{\text{mean}\left(D_{\text{yyproj}},D_{\text{zzassoc}}\right)}$$

### 2.6 Statistical analysis

Demographic and clinical characteristics were compared among the three groups using the SPSS software (version 27). Categorical variables were expressed as counts (percentages) and analyzed by the chi-square test. The normality test was assessed using the Shapiro-Wilk test. Continuous data were expressed as mean ± standard deviation (SD) for normally distributed variables. One-way analysis of variance (ANOVA) was applied to compare the overall differences among the CU, SCD, and MCI groups. Post hoc tests with Bonferroni correction were performed to investigate which pairs of groups differed after an overall difference had been established. Subsequently, analysis of covariance (ANCOVA) was employed to compare differences in the ALPS index and PVS burden among the CU, SCD, and MCI groups, with age, gender, and education level as covariates. After controlling for age, sex, and years of education, partial correlation analysis was used to assess the relation between neuroimaging metrics and cognitive function. The results were corrected for multiple comparisons using the false discovery rate(FDR) method. The intraclass correlation coefficient (ICC) was used to assess the inter-observer agreement. All significance tests were two-tailed, and a *p*-value < 0.05 was considered statistically significant.

## 3 Results

### 3.1 Participants characteristics

A comprehensive summary of the baseline demographics and clinical features of the participant cohort is provided in Table 1. The study cohort comprised 126 participants, consisting of 21 CU (11F/10M, age 65.14 ± 8.49 years), 68 patients with SCD (37F/31M, age 63.53 ± 6.83 years), and 37 patients with MCI (28F/9M, age 64.24 ± 7.48 years). The MCI and SCD groups were less educated than the CU group (*p* < 0.001). There was no significant difference in age, sex, hypertention and diabetes.

**Table 1:** Demographic and clinical characteristics of the participants.

	CU (n= 21)	SCD (n= 68)	MCI (n= 37)	P-value
Age,years	65.14 ± 8.49	63.53 ± 6.83	64.24 ± 7.48	0.660
Sex,female,N(%)	11 (52%)	37 (54%)	28 (75%)	0.075
Education, years	13.90 ± 2.84	11.49 ± 2.55	10.35 ± 2.37	< 0.001⁎⁎⁎
Hypertention,N(%)	10 (48%)	34 (50%)	14 (38%)	0.484
Diabetes,N(%)	2 (10%)	15 (22%)	8 (22%)	0.430
MMSE	28.48 ± 1.25	27.85 ± 1.93	26.05 ± 2.29	< 0.001⁎⁎⁎
MoCA	26.48 ± 2.18	23.54 ± 2.69	20.92 ± 2.79	< 0.001⁎⁎⁎
TMTA	64.48 ± 23.46	59.43 ± 17.64	82.62 ± 27.67	< 0.001⁎⁎⁎
TMTB	134.52 ± 44.31	153.50 ± 42.19	216.32 ± 59.22	< 0.001⁎⁎⁎
AFT	20.24 ± 4.89	16.69 ± 4.52	14.49 ± 3.85	< 0.001⁎⁎⁎
BNT	24.67 ± 4.52	25.31 ± 3.07	22.89 ± 3.94	0.005⁎⁎
DST	18.05 ± 3.25	16.14 ± 4.16	15.22 ± 3.08	0.023*
SDMT	41.65 ± 10.70	34.58 ± 9.28	27.72 ± 14.64	< 0.001⁎⁎⁎
WSL	19.24 ± 6.07	13.46 ± 6.43	9.76 ± 4.59	< 0.001⁎⁎⁎
AVLT				
N5	5.85 ± 2.82	4.88 ± 1.83	2.70 ± 1.79	< 0.001⁎⁎⁎
N7	21.62 ± 1.52	20.69 ± 2.05	18.14 ± 3.17	< 0.001⁎⁎⁎
N1	4.62 ± 2.09	2.90 ± 1.38	2.11 ± 1.33	< 0.001⁎⁎⁎
N2	6.43 ± 2.11	5.49 ± 1.65	4.54 ± 1.28	< 0.001⁎⁎⁎
N3	7.33 ± 1.88	6.62 ± 1.62	5.65 ± 1.38	< 0.001⁎⁎⁎
N4	6.05 ± 3.04	5.29 ± 1.69	3.14 ± 1.87	< 0.001⁎⁎⁎
N6	6.05 ± 2.78	5.06 ± 2.00	3.11 ± 1.75	< 0.001⁎⁎⁎
Aβ 1-42	48.85 ± 29.33(12)	88.31± 68.09(53)	102.51 ± 141.47(33)	0.264
Tau-181	10.09 ± 10.73(12)	16.55 ± 9.92(53)	20.21 ± 27.56(33)	0.245
CHOL	4.81 ± 1.00(19)	5.08 ± 0.98(61)	5.65 ± 1.19(37)	0.007⁎⁎
TG	1.42 ± 0.82(19)	1.45 ± 0.82(62)	1.60 ± 0.78(37)	0.609
HDL-C	1.30 ± 0.37(19)	1.39 ± 0.31(62)	1.49 ± 0.26(37)	0.084
LDL-C	2.97 ± 0.85(19)	3.12 ± 0.79(61)	3.54 ± 0.87(37)	0.017*
APOB	0.90 ± 0.26(19)	1.05 ± 0.25(62)	1.20 ± 0.31(37)	0.001⁎⁎
GLU	6.03 ± 1.76(10)	5.57 ± 1.09(62)	6.41 ± 2.80(37)	0.102
Fazekas score total	2.21 ± 1.03	2.10 ± 1.07	2.70 ± 1.29	0.033*
PVWM	1.25 ± 0.65	1.34 ± 0.59	1.49 ± 0.69	0.301
DWM	0.96 ± 0.51	0.76 ± 0.65	1.22 ± 0.71	0.003⁎⁎

Results are expressed as means ± standard deviation for the continuous variables and as frequencies for the categorical variables.

Abbreviations: CU, cognitive unimpaired; SCD, subjective cognitive decline; MCI, mild cognitive impairment; MMSE, mini-mental status examination; MoCA, Montreal cognitive assessment; TMT A, trail making test A; TMT B, trail making test B; AFT, animal fluency test; BNT, Boston naming test; DST, Digit Span Task; SDMT, the Symbol Digit Modalities Test; WSL, Logical memory of Wechsler Memory Scale; AVLT, auditory verbal learning test; Aβ, amyloid beta; Tau, phosphorylated tau protein; CHOL, the total cholesterol; TG, the triglyceride; HDL-C, the high-density lipoprotein; LDL-C, the low-density lipoprotein; APOB, the apolipoprotein; GLU, the blood glucose; PVWM periventricular white matter; DWM, deep white matter.

Group comparisons were done with the one-way ANOVA (quantitative variables) and chi-square test (categorical variables).

⁎ indicates p value < 0.05

⁎⁎ indicates p value < 0.01

⁎⁎⁎ indicates p value < 0.001.

Significant differences in Mini-Mental State Examination (MMSE), Montreal Cognitive Assessment (MoCA), and other neuropsychological test scores were identified across the three groups (*p* < 0.001). Compared with CU participants, both the SCD and MCI groups performed significantly worse on Trail Making Tests A and B (TMTA, TMTB), Animal Fluency Test (AFT), Boston Naming Test (BNT), Digit Span Test (DST), Symbol Digit Modalities Test (SDMT), Word Span List (WSL), delayed recall (AVLT-N5),recognition(AVLT-N7), and additional cognitive subtests (AVLT-N1 through AVLT-N4 and AVLT-N6), as well as on global cognition measures (MMSE and MoCA).

Significant between-group differences were observed for the serological indicators CHOL (*p =* 0.007), LDL-C (*p =* 0.02), and APOB (*p* < 0.001), while no significant differences were observed in the others, including TG, HDL-C, GLU, Aβ 1-42 and Tau-181. Furthermore, significant differences were observed in the deep WM hyperintensities and total Fazekas scores (*p* = 0.003 and *p =* 0.03, respectively).

**Agreement analysis of EPVS visual grading and ALPS calculation**

The inter-rater agreement for PVS visual grading between two readers was good (ICC range: 0.820–0.851). For the placement of regions of interest in ALPS calculation, the consistency between two researchers was excellent (ICC range: 0.931–0.964). The specific data are presented in Supplementary S2(1).

### 3.2 Increased PVS-BG burden in patients with MCI

**Fig. 3:**
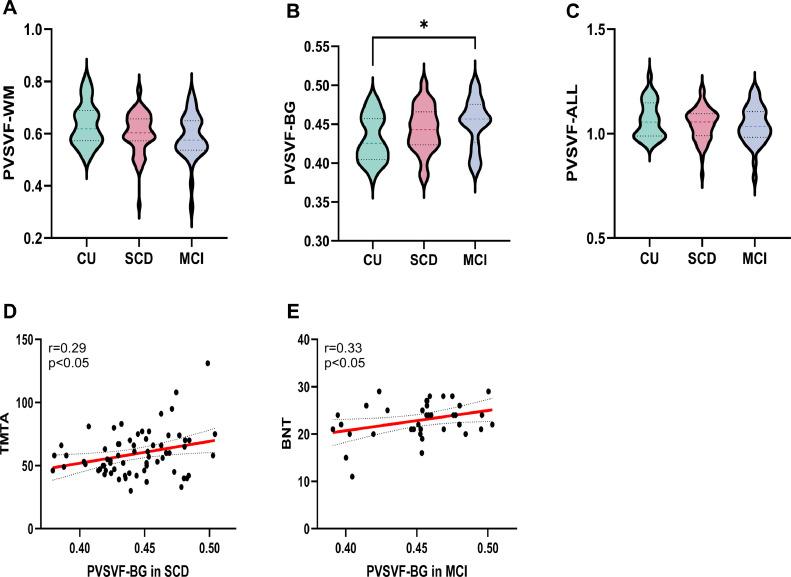
Group differences in PVSVF and its relationship with cognition Fig. 3 Differences in PVS volume fraction across the CU, SCD, and MCI groups (A–C), along with relationships between PVS volume fraction and cognitive measures that were significantly correlated in Pearson correlation analyses within the SCD and MCI cohorts. The red line represents a linear regression with a 95% confidence interval (dashed line)(D-E). WM, white matter; BG,basal ganglia; PVSVF, perivascular spaces volume fraction; CU, cognitive unimpaired; SCD, subjective cognitive decline; MCI, mild cognitive impairment; TMT A, trail making test A; BNT, Boston naming test; *p < 0.05, statistically significant.

The group comparisons of MRI-visible PVS and PVS volume fraction of CU, SCD, and MCI groups summarizes in Table 2. Among all measured parameters, only PVSVF-BG demonstrated statistically significant intergroup variations (*p* = 0.03). Post-hoc multiple comparison analysis revealed that patients with MCI exhibited significantly higher PVSVF-BG values than CUs (*p* = 0.02) (Fig. 3 B)

**Table 2:** Group comparisons of MRI-visible PVS andPVS volume fraction of CU, SCD,and MCI groups.

	CU	SCD	MCI	P value
CSO-PVS grade	28	68	37	0.209
Grade0,n	4	3	0	
Grade1,n	61	28	26	
Grade2,n	7	23	9	
Grade3,n	1	4	2	
Grade4,n	0	0	0	
WM-PVS,%	0.64 ± 0.08	0.60 ± 0.08	0.59 ± 0.09	0.062
BG-PVS grade	28	68	37	0.215
Grade0,n	0	1	0	
Grade1,n	19	43	17	
Grade2,n	9	23	17	
Grade3,n	0	1	3	
Grade4,n	0	0	0	
BG-PVS,%	0.43 ± 0.03	0.44 ± 0.03	0.45 ± 0.03	0.029*
Midbrain grade	28	68	37	0.445
0, n	20	41	26	
1, n	8	27	11	

Results are expressed as means ± standard deviation for the continuous variables and as frequencies for the categorical variables.

Abbreviations: CU, cognitive unimpaired; SCD, subjective cognitive decline; MCI, mild cognitive impairment; CSO, centrum semiovale; BG, basal ganglia; PVS, perivascular spaces.

Group comparisons were done with the one-way ANOVA (quantitative variables) and chi-square test (categorical variables).

⁎ indicates p value < 0.05.

### 3.3 Decreased ALPS in patients with MCI and SCD

**Fig. 4:**
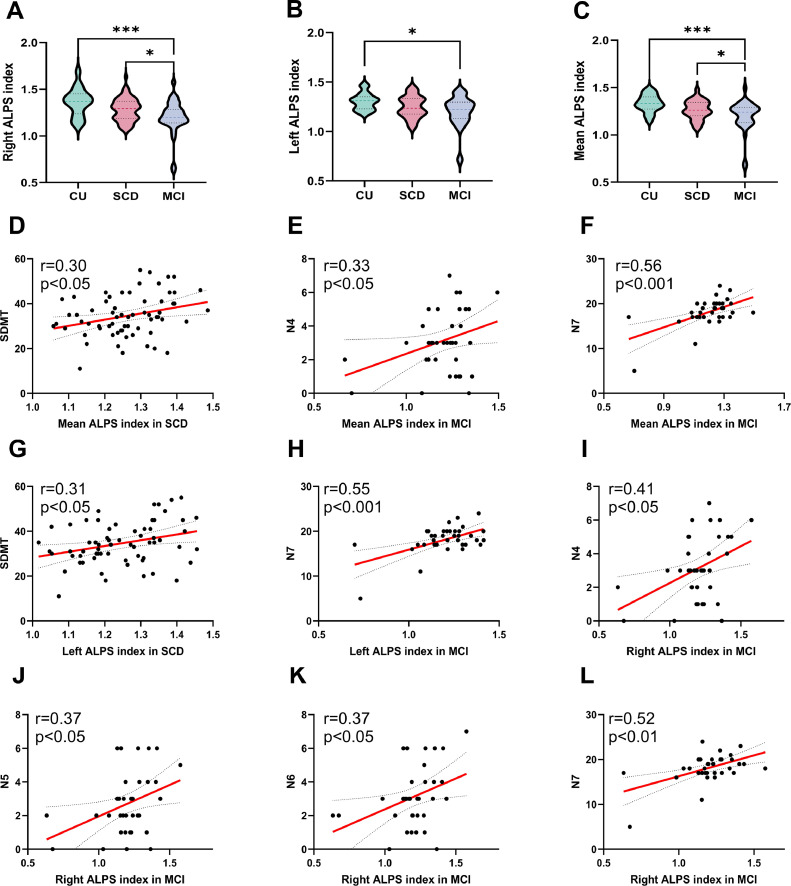
Group differences in DTI-ALPS index and its relationship with cognition Fig. 4 Differences in DTI-ALPS index among CU, SCD, and MCI groups (A–C), along with relationships between DTI-ALPS index and cognitive measures that were significantly correlated in Pearson correlation analyses within the SCD and MCI cohorts. The red line represents a linear regression with a 95% confidence interval (dashed line)(D-L). ALPS, diffusion tensor image analysis along the perivascular space; CU, cognitive unimpaired; SCD, subjective cognitive decline; MCI, mild cognitive impairment; SDMT, the Symbol Digit Modalities Test; AVLT, auditory verbal learning test; *p < 0.05, ***p < 0.001, statistically significant.

The group comparisons of ALPS index among the CU, SCD, and MCI groups shows in Table 3. Significant intergroup differences were observed across all three groups for both right (*p* < 0.001) and left (*p =* 0.02) ALPS indices, as well as for whole-brain mean ALPS values (*p* < 0.001). Significant differences in diffusion metrics were also found among the CU, SCD, and MCI groups, including right hemispheric Dxassoc (*p* = 0.049), Dzassoc (*p =* 0.02), and Dyproj (*p =* 0.01), and left hemispheric Dxproj (*p =* 0.04), Dzassoc (*p =* 0.01), and Dyproj (*p =* 0.007). Post-hoc analysis revealed that patients with MCI exhibited significantly lower values than CU in right ALPS index (*p* < 0.001), left ALPS index (*p =* 0.01), and mean ALPS index (*p* < 0.001). Additionally, the MCI group showed significantly lower right ALPS index (*p =* 0.02) and mean ALPS index (*p =* 0.03) compared to SCD (Fig. 4 A-C).

**Table 3:** Group comparisons of DTI-ALPS index of CU,SCD,and MCI groups.

	CU (n = 21)	SCD (n = 68)	MCI (n = 37)	P-value
Right DTI-ALPS index	1.36 ± 0.15	1.28 ± 0.12	1.20 ± 0.18	< 0.001⁎⁎⁎
Right Dxassoc, × 10^-3^	0.62 ± 0.14	0.70 ± 0.13	0.68 ± 0.12	0.049*
Right Dxproj, × 10^-3^	0.55 ± 0.08	0.61 ± 0.09	0.63 ± 0.15	0.062
Right Dzassoc, × 10^-3^	0.35 ± 0.15	0.43 ± 0.14	0.48 ± 0.21	0.020*
Right Dyproj, × 10^-3^	0.52 ± 0.06	0.60 ± 0.10	0.64 ± 0.20	0.010*
Left DTI-ALPS index	1.30 ± 0.08	1.25 ± 0.11	1.20 ± 0.16	0.017*
Left Dxassoc, × 10^-3^	0.76 ± 0.06	0.81 ± 0.07	0.79 ± 0.12	0.076
Left Dxproj, × 10^-3^	0.60 ± 0.06	0.64 ± 0.06	0.65 ± 0.10	0.036*
Left Dzassoc, × 10^-3^	0.49 ± 0.05	0.55 ± 0.10	0.59 ± 0.14	0.010*
Left Dyproj, × 10^-3^	0.56 ± 0.06	0.61 ± 0.07	0.64 ± 0.12	0.007⁎⁎
Mean DTI-ALPS index	1.33 ± 0.09	1.26 ± 0.10	1.20 ± 0.16	< 0.001⁎⁎⁎

Results are expressed as means ± standard deviation for the continuous variables.

Abbreviations: CU, cognitive unimpaired; SCD, subjective cognitive decline; MCI, mild cognitive impairment. Right DTI-ALPS index, right-hemispheric diffusion tensor image analysis along the perivascular space; Left DTI-ALPS index,left-hemispheric DTI-ALPS; Mean DTI-ALPS index, the mean of the sum of right-hemispheric DTI-ALPS and left-hemispheric DTI-ALPS; Dxassoc, diffusivities along the x-axis of ROIs within association fibers; Dxproj, diffusivities along the x-axis of ROIs within projection fibers; Dzassoc, diffusivities along the z-axis of ROIs within association fibers; Dyproj, diffusivities along the y-axis of ROIs within projection fibers. One-way ANOVA was applied in normally distributed continuous data

⁎ indicates p value < 0.05

⁎⁎ indicates p value < 0.01

⁎⁎⁎ indicates p value < 0.001.

### 3.4 Correlations of MRI indices with clinical characteristics

Correlation analyses between the MRI **indices** and clinical characteristics were conducted separately in the SCD and MCI groups. Notably, in SCD, a higher PVSVF-BG was associated with poorer performance on the TMTA (*r* =0.290, *p* = 0.016, *p* = 0.256 after FDR corrected), whereas an elevated PVSVF-BG showed a significant correlation with scores on the BNT in MCI (*r* = 0.339, *p* = 0.040, *p* = 0.520 after corrected)(Fig. 3D-E). Regarding glymphatic function, both the mean (r = 0.295, p = 0.017, p = 0.272 after corrected) and left-side ALPS indices (r = 0.311, p = 0.012, p = 0.192 after corrected) exhibited positive correlation with information processing speed (SDMT scores) in the SCD group. Specifically within the MCI group, higher mean, left-side and right-side ALPS indices were significantly associated with superior episodic memory performance (as measured by N4, N5, N6, and N7 scores on the AVLT)(Fig. 4D-L). Although most correlations between AVLT subscores and ALPS indices were non-significant after FDR correction, the associations with the N7 delayed recall subscore remained robust to FDR correction, including mean ALPS (r = 0.564, p = 0.004 after corrected), left-side ALPS (r = 0.551, p = 0.006 after corrected), and right-side ALPS (r = 0.519, p = 0.016 after corrected). This pattern suggests that glymphatic function may be more closely linked to attention in the early disease stage (SCD), whereas its association with memory function becomes more prominent at the MCI stage. No significant correlations were observed between imaging biomarkers (ALPS and PVS), nor between imaging biomarkers and serum biomarkers (Aβand Tau), in either SCD or MCI, as shown in Supplementary S2(2-4).

## 4 Discussion

SCD and MCI represent high-risk populations for AD and are widely regarded as key intervention stages for dementia. Given the limited research on the correlation between the ALPS index and PVS burden in the preclinical stage of AD, this study employs non-invasive MRI metrics to investigate their glymphatic system function. The findings reveal that MCI patients exhibit increased PVSVF-BG compared to CUs, and both the ALPS indices in the left and right hemispheres are significantly reduced. Furthermore, the ALPS indices in the right hemisphere and the whole brain of MCI patients is lower than that of SCD patients. These indicators suggest dilation of PVS and reduced water diffusivity along venular perivascular spaces, likely resulting from impaired glymphatic function. Moreover, this impairment appears more severe in MCI than in SCD or CU. Additionally, we found that indicators of glymphatic dysfunction were correlated with impaired performance in cognitive domains including attention and memory.

Compared to CUs, we did not observe significantly higher PVSVF-BG in individuals with SCD, nor were any significant differences detected among the three groups through qualitative analysis. However, a elevated PVSVF-BG was observed in MCI. Previous studies have reported higher PVSVF-BG in AD patients compared to cognitively normal individuals [13,28], which aligns with the trend observed in our findings. Studies have also indicated that compared to CU subjects, individuals with MCI and AD exhibited higher numbers, grades, and volume fractions of CSO-PVS [28,29]. This phenomenon may be attributed to multiple factors, including age, white matter hyperintensity volume, and amyloid and tau pathology. Notably, a high burden of MR-visible perivascular spaces in the centrum semiovale is independently associated with β-amyloid positivity [29]. Additionally, a longitudinal study observed an accelerated rate of change in CSO-PVS burden in amyloid-positive individuals, while combined amyloid and tau positivity was associated with accelerated progression of PVS-BG burden [30]. These findings collectively indicate that EPVS volume is closely associated with aging and Alzheimer's disease pathology. Specifically, the accumulation of cerebral waste products such as Aβ and tau proteins may impede perivascular drainage of interstitial fluid, ultimately leading to impaired clearance and dilation of perivascular spaces. As the earliest preclinical symptomatic stage of AD, structural brain changes in SCD are typically subtle. The lack of a significant increase in PVS burden in the SCD group may suggest that, at this very early disease stage, the glymphatic system remains partially compensatory, or that PVS dilation has not yet reached the threshold for detection by conventional MRI. By the time cognitive impairment meets the objective diagnostic criteria for MCI, prolonged clearance dysfunction may have already led to structural remodeling of the PVS.

The core finding of this study is that the ALPS index begins to decline at the SCD stage and shows a further significant decrease at the MCI stage. A decreased ALPS index may suggest impaired glymphatic function [31]. Compared with the CU and SCD groups, our study found that MCI patients exhibited a lower ALPS index in both individual cerebral hemispheres and across the whole brain. This suggests that the ALPS index holds promise as a non-invasive biomarker for glymphatic impairment in the early symptomatic stages of AD. Previous studies have shown that the ALPS index is significantly lower in patients with AD and MCI, which is consistent with our findings [19,32]. Likewise, a case-control study revealed diminished glymphatic activity in the MCI group relative to control cohorts [33]. Another study, however, reported no significant differences between MCI patients and controls [28]. This discrepancy may be explained by the study’s small sample size and differences in participants educational backgrounds. Recent studies further highlight that a decline in the ALPS index correlates with a greater risk for MCI and AD [28,31,34]. Moreover, research focusing on SCD has revealed a progressive decline in the ALPS index throughout the AD continuum, starting from SCD to MCI and advancing to AD dementia recently [35]. These findings imply that glymphatic dysfunction may emerge in the earlier phases of Alzheimer's disease. Meanwhile, studies have also indicated that the ALPS index begins to decline as early as the SCD stage [36]. The reduction is particularly pronounced in MCI and differs significantly from that in the SCD group, suggesting that the progressive deterioration of glymphatic function may parallel the transition from subjective cognitive complaints to objective cognitive deficits.

The correlation between the ALPS index and cognitive domains shifts across disease stages, degenerating processing speed in SCD and to memory in MCI. A more efficient glymphatic system generally predicts better cognitive performance. This clinical association reveals a close pathophysiological link between the glymphatic system, the metabolism of toxic proteins, and cognitive impairment. The core mechanism lies in the regulatory role of the glymphatic system in clearing Aβ and Tau [37]. Existing research has confirmed that glymphatic activity serves as a key mediator between the deposition of Aβ and Tau in multiple brain regions and the corresponding cognitive dysfunction [38]. In the earliest disease stage (SCD), glymphatic system dysfunction may initially impair cognitive domains requiring rapid information integration. As the disease progresses to MCI, key memory-related brain regions such as the medial temporal lobe accumulate more pathological protein deposits. Therefore, impairments in specific cognitive domains at different disease stages may reflect the spatiotemporal evolution of Alzheimer's disease pathology, providing critical insights for early detection and targeted interventions. EPVS burden reflects structural changes, while the ALPS index reflects functional status. No significant correlation was observed between the two measures. However, glymphatic system dysfunction may be a gradual process in which functional impairment precedes structural alterations. During the SCD and MCI stages, dynamic function may have already begun to decline, whereas anatomical dilation might not yet be apparent.

To develop simpler and more practical diagnostic tools for populations at high risk of AD, we collected plasma biomarker measurements. Recent evidence supports plasma p-tau181 as a useful biomarker that helps distinguish Aβ-positive SCD, from Aβ-positive CU individuals, and aids in identifying those at risk of early cognitive decline [39]. Moreover, p-tau181 shows promise as a non-invasive marker of very early AD pathology [40]. Differences in plasma biomarker levels among SCD, MCI, and AD dementia patients appear to reflect underlying pathology rather than cognitive performance [41]. Glymphatic function, measured via the ALPS index, negatively correlates with plasma p-tau levels and mediates the association between p-tau and cognition [42]. Regarding Aβ, AD patients show reduced plasma Aβ42 levels and Aβ42/40 ratio, while Aβ-PET-positive SCD patients exhibit elevated Aβ42 and Aβ40 [43]. Our study found no significant differences in plasma biomarkers (Aβ42 and tau181) across the three groups, nor were they associated with metrics of glymphatic function either. However, this should not discount their diagnostic potential in high-risk AD cohorts, as the limited sample size and lack of stratification by pathological biomarker status may have affected the results. Future work will involve larger samples to clarify the diagnostic utility of these plasma markers in high-risk populations, combined with glymphatic imaging and cognitive assessments to improve predictive models for AD.

This study has several limitations. First, the sample size was relatively small; future studies should include larger cohorts. Second, as this was a cross-sectional study, longitudinal designs are needed to follow both high-risk AD populations and cognitively unimpaired individuals over time, tracking changes in glymphatic function and cognitive performance. Third, glymphatic function may be influenced by circadian rhythms and sleep quality. It is necessary to further investigate the effects of different scanning timepoints and varying sleep quality on glymphatic activity. Besides, the absence of cerebrospinal fluid or PET biomarkers hinders precise assessment of cerebral Aβ and tau burden. Finally, the relationship between DTI-ALPS and the glymphatic system has not been evaluated through pathophysiological studies, and should therefore be interpreted with caution [44]. Future research should integrate multimodal data and utilize methods such as gBOLD-CSF coupling and functional connectivity to achieve a more comprehensive analysis of the glymphatic system in high-risk AD populations, thereby improving early identification of high-risk individuals, and facilitating timely intervention.

## 5 Conclusions

This study confirms that glymphatic system dysfunction (reduced ALPS index) and local perivascular space enlargement (increased PVS burden in the basal ganglia) are early imaging features present across the AD risk spectrum, with their association to cognitive function demonstrating disease-stage specificity. These findings underscore the central role of the brain’s fluid clearance system in the early pathological processes of AD and highlight the importance of targeting this system for monitoring disease progression and developing potential therapeutic interventions.

## Fundings

This work was supported by the National Key R&D Program of China (2023YFC3603605), the Science & Technology Department of Jiangsu Province (BE2023778), the 10.13039/501100001809National Natural Science Foundation of China (82401788), the Jiangsu Provincial Natural Science Youth Fund (BK20241120) and Liyang City's 2023 Annual research and development Plan Follows Nanjing Project (LC2024001).

## Ethics statement

The patients/participants provided their written informed consents to participate in this study. All procedures performed in studies involving human participants were in accordance with the ethical standards of the institutional and/or national research committee and with the 1964 Helsinki declaration and its later amendments or comparable ethical standards. The studies involving human participants were reviewed and approved by The Ethics Committee of the First Affiliated Hospital of Nanjing Medical University in April 2019 (2019-SR-015).

## Data statement

The datasets generated and analyzed during the current study are available from the corresponding author on reasonable request.

## Declaration of the use of generative AI and AI-assisted technologies in scientific writing and in figures, images and artwork

We did not use generative AI to generate any manuscript text, but help polish some sentences.

## Data sharing statement

The codes and dataset are available from the corresponding author upon reasonable request.

## CRediT authorship contribution statement

**Li Jiang:** Writing – original draft, Methodology, Data curation, Conceptualization. **Ling Zhang:** Writing – original draft, Data curation, Conceptualization. **Shu-Xian Wu:** Investigation, Formal analysis, Data curation. **Qin-Qin Zhu:** Validation, Software, Data curation. **Wei Wang:** Methodology, Conceptualization. **Jia-Wei Gao:** Data curation. **Yi Zhu:** Resources, Funding acquisition. **Shui Tian:** Writing – review & editing, Supervision, Project administration, Methodology, Funding acquisition. **Ming Qi:** Writing – review & editing, Supervision, Resources, Project administration, Funding acquisition.

## Declaration of interest

All authors report no conflicts of interests.

## Appendix Supplementary materials

Image, application 1

Image, application 2
