# Supplementary material for: Association between MRI indicators of the glymphatic system and cognition in high-risk populations for Alzheimer's disease
Source: J Prev Alzheimers Dis. 2026 Feb 20;13(4):100504. doi: 10.1016/j.tjpad.2026.100504 (PMC12934294; doi:10.1016/j.tjpad.2026.100504)
Supplement: Supplementary file 1 [file mmc1.docx]

**SUPPLEMENTARY S1**

**Supplementary 1**

At the time of participant recruitment for this study, the following neuropsychological assessments were administered: Global cognitive function was initially evaluated in all participants using the Mini-Mental State Examination (MMSE) and Montreal Cognitive Assessment (MoCA)—these instruments demonstrate high sensitivity and specificity in distinguishing mild cognitive impairment (MCI) from healthy controls (HCs) and dementia populations. Furthermore, a comprehensive neuropsychological test battery was implemented to assess four cognitive domains: (1) Memory function was evaluated using the Auditory Verbal Learning Test (AVLT) and the Logical Memory subtest of the Wechsler Memory Scale (WMS-LM); (2) Executive function was assessed via Trail Making Test Parts A and B (TMT-A and TMT-B); (3) Language function was examined using the Boston Naming Test (BNT) and Animal Fluency Test (AFT); and (4) Attention function was measured using the Digit Span Task (DST) and the Symbol Digit Modalities Test (SDMT).

**Supplementary 2**

Diffusion Tensor Imaging (DTI), T1-weighted (T1w), and fluidattenuated inversion recovery (FLAIR) imaging data were acquired for each participant using a 3.0 T-MR scanner (750 W, GE Healthcare, USA) with a 24-channel head coil.

T1-weighted anatomic images were obtained using a 3D BRAVO sequence based on fast rotating gradient echo with the following parameters: field of view (FOV) = 256 × 256 mm2 ; repetition time (TR) = 8.5 ms, echo time (TE) = 3.2 ms, inversion time (TI) = 450 ms, flip angle (FA) = 12; slice thickness = 1 mm; and acquisition matrix = 256 × 256.

FLAIR images was obtained with the following parameters: FOV= 240×240 mm2 ; TR= 8000 ms, TE = 95 ms, FA = 160 ; slice thickness = 5 mm; and acquisition matrix =256×254.

DTI data acquisition parameters were set as follows: b-values of b=0 and b=1000 s/mm²; 30 diffusion encoding directions; field of view (FOV) = 224 × 224 mm²; TR = 8396 ms, TE was set to the minimum achievable value; slice thickness = 3 mm; acquisition matrix = 112 × 112.
