# Supplementary material for: Association between MRI indicators of the glymphatic system and cognition in high-risk populations for Alzheimer's disease
Source: J Prev Alzheimers Dis. 2026 Feb 20;13(4):100504. doi: 10.1016/j.tjpad.2026.100504 (PMC12934294; doi:10.1016/j.tjpad.2026.100504)
Supplement: Supplementary file 2 [file mmc2.docx]

**SUPPLEMENTARY S2**

|  | ICC(95% CI) | P-value |
| --- | --- | --- |
| COS-PVS grade | 0.820 (0.656, 0.910) | < 0.001 |
| BG-PVS grade | 0.851 (0.711, 0.926) | < 0.001 |
| Midbrain grade | 0.840 (0.691, 0.921) | < 0.001 |
| Right DTI-ALPS index | 0.964 (0.926, 0.983) | < 0.001 |
| Left DTI-ALPS index | 0.956 (0.909, 0.979) | < 0.001 |
| Mean DTI-ALPS index | 0.931 (0.861, 0.967) | < 0.001 |

**Supplementary (1)** Agreement analysis of EPVS visual grading and ALPS calculation

CSO, centrum semiovale; BG, basal ganglia; PVS, perivascular spaces.

Right DTI-ALPS index, right-hemispheric diffusion tensor image analysis along the perivascular space; Left DTI-ALPS index ,left-hemispheric DTI-ALPS; Mean DTI-ALPS index, the mean of the sum of right-hemispheric DTI-ALPS and left-hemispheric DTI-ALPS

**Supplementary (2)** Correlation of imaging biomarkers PVS and ALPS.


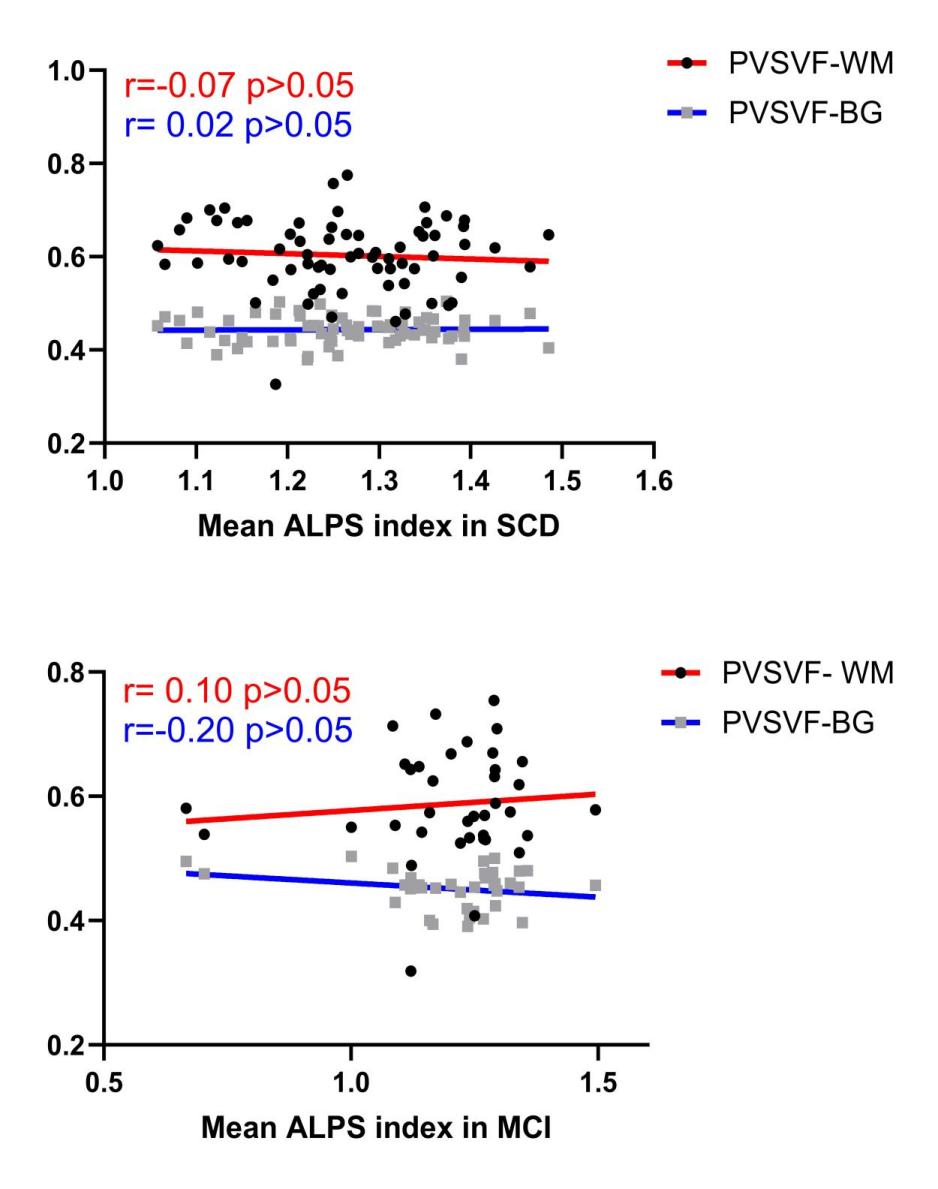


WM, white matter; BG,basal ganglia; PVSVF, perivascular spaces volume fraction; SCD, subjective cognitive decline; MCI, mild cognitive impairment; ALPS, diffusion tensor image analysis along the perivascular space.

**Supplementary (3)** Correlation between the imaging biomarkers PVS and ALPS with the serological biomarkers Aβ and Tau in SCD.


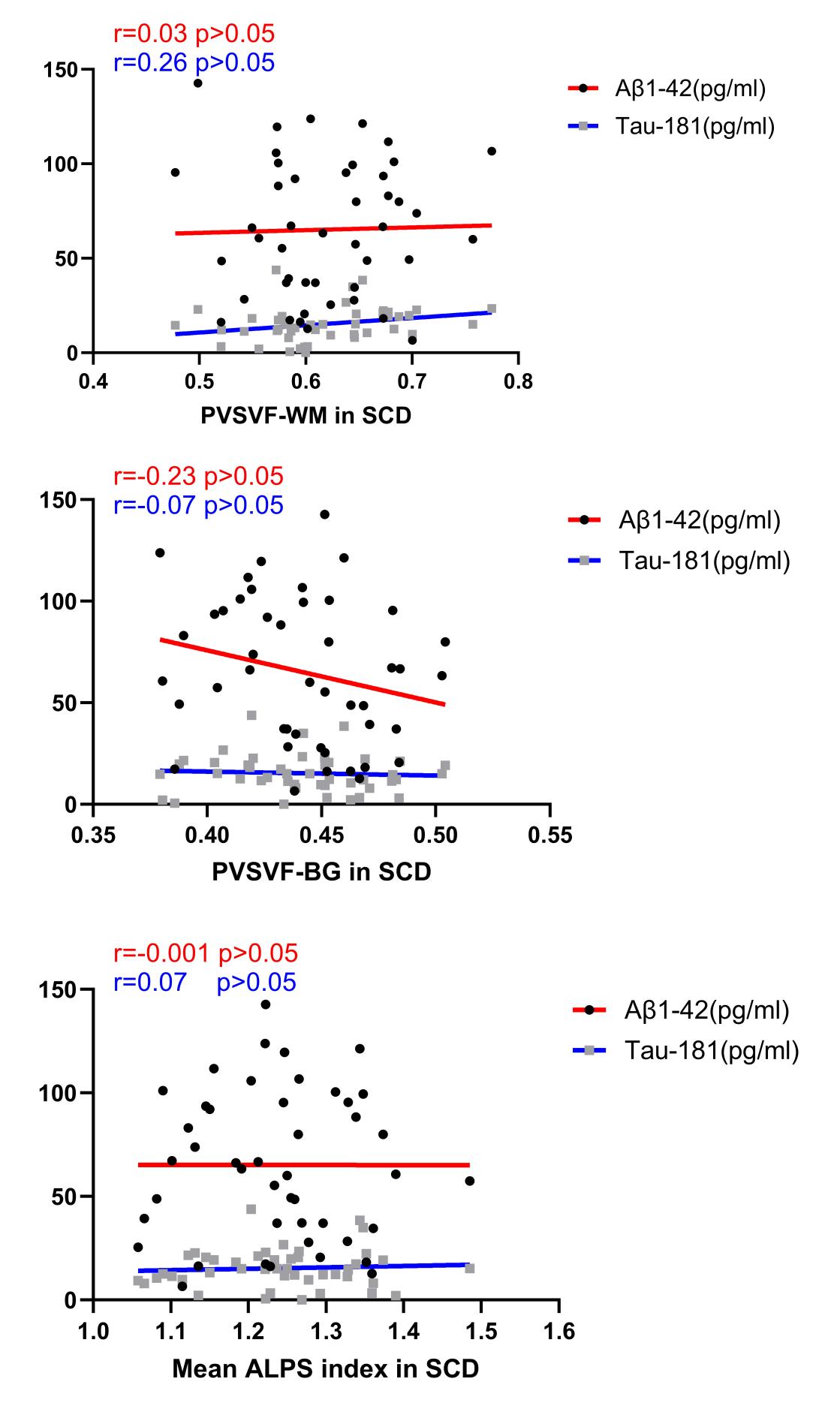


WM, white matter; BG,basal ganglia; PVSVF, perivascular spaces volume fraction; SCD, subjective cognitive decline; ALPS, diffusion tensor image analysis along the perivascular space; Aβ, amyloid beta; Tau, phosphorylated tau protein

**Supplementary (4)** Correlation between the imaging biomarkers PVS and ALPS with the serological biomarkers Aβ and Tau in MCI.


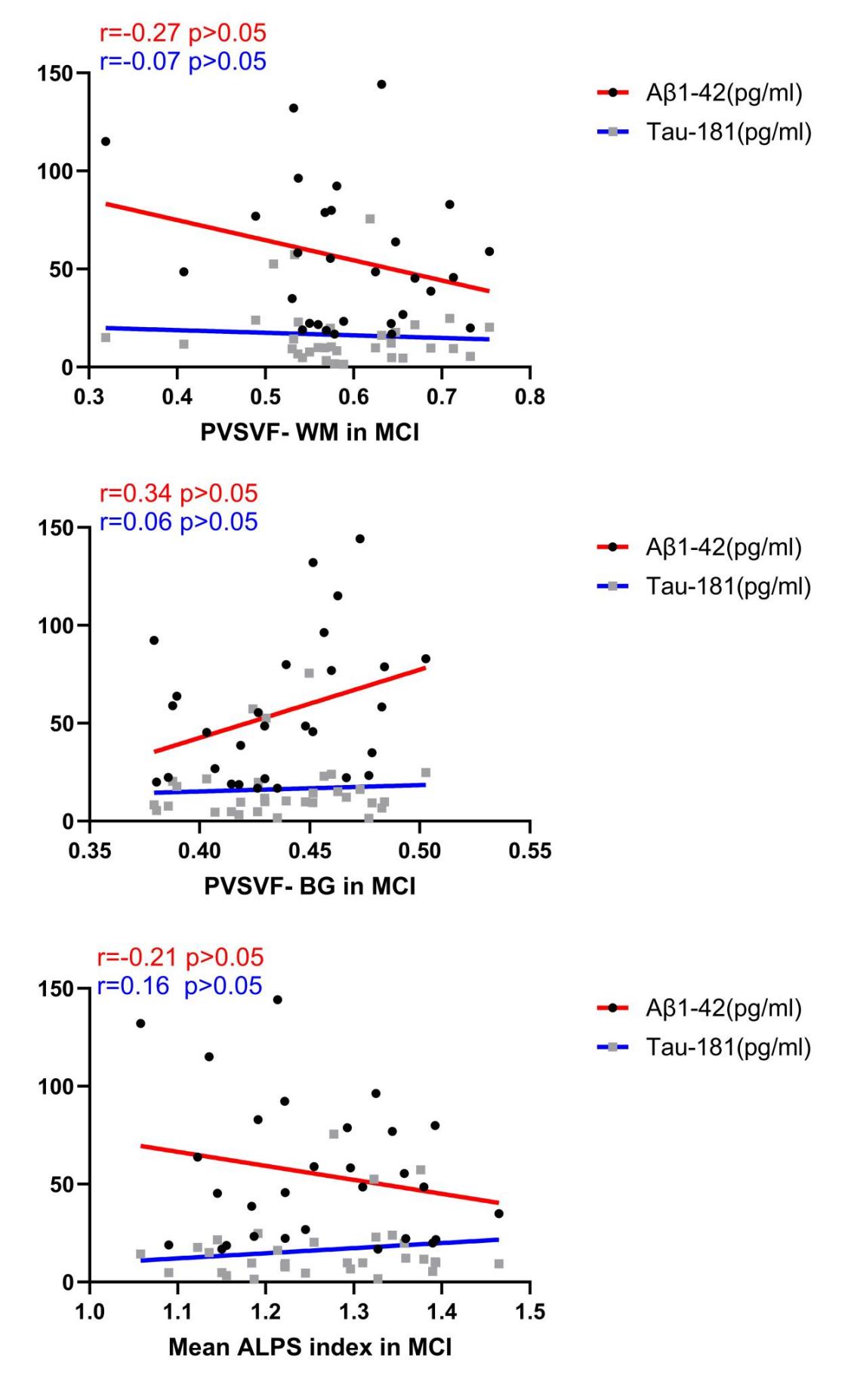


WM, white matter; BG,basal ganglia; PVSVF, perivascular spaces volume fraction; MCI, mild cognitive impairment; ALPS, diffusion tensor image analysis along the perivascular space; Aβ, amyloid beta; Tau, phosphorylated tau protein
